# Correction: A family of fluoride-specific ion channels with dual-topology architecture

**DOI:** 10.7554/eLife.01700

**Published:** 2013-10-22

**Authors:** Randy B Stockbridge, Janice L Robertson, Ludmila Kolmakova-Partensky, Christopher Miller

Stockbridge RB, Robertson JL, Kolmakova-Partensky L, Miller C. 2013. A family of fluoride-specific ion channels with dual-topology architecture. *eLife* **2**:e01084. doi: 10.7554/eLife.01084. Published 27 August 2013

In Figure 1A, the 3rd and 4th sequences and the 12th and 13th sequences were incorrectly labeled as La1 and La2. The 3rd and 12th sequences should have been La2, and the 4th and 13th sequences, La1.

The article has now been corrected.

